# Identification of quantitative trait loci underlying resistance to southern root-knot and reniform nematodes in soybean accession PI 567516C

**DOI:** 10.1007/s11032-015-0330-5

**Published:** 2015-05-23

**Authors:** Yongqing Jiao, Tri D. Vuong, Yang Liu, Zenglu Li, Jim Noe, Robert T. Robbins, Trupti Joshi, Dong Xu, J. Grover Shannon, Henry T. Nguyen

**Affiliations:** Division of Plant Sciences and National Center for Soybean Biotechnology (NCSB), University of Missouri, Columbia, MO 65211 USA; Computer Science Department and Christopher S Bond Life Sciences Center, Informatics Institute, University of Missouri, Columbia, MO 65211 USA; Center for Applied Genetic Technologies and Department of Crop and Soil Sciences, University of Georgia, Athens, GA 30602 USA; Department of Plant Pathology, University of Georgia, Athens, GA 30602 USA; Department of Plant Pathology, University of Arkansas, Fayetteville, AR 73701 USA; Division of Plant Sciences and National Center for Soybean Biotechnology (NCSB), University of Missouri, Delta Center, P.O. Box 160, Portageville, MO 63873 USA; Key Laboratory of Oil Crop Biology of the Ministry of Agriculture, Oil Crops Research Institute of the Chinese Academy of Agricultural Sciences, Wuhan, 430062 Hubei China

**Keywords:** Southern root-knot nematode, Reniform nematode, QTL, Soybean, PI 567516C

## Abstract

Soybean cyst nematode (SCN, *Heterodera glycine* Ichinohe), southern root-knot nematode [SRKN, *Meloidogyne incognita* (Kofoid and White) Chitwood] and reniform nematode (RN, *Rotylenchulus reniformis* Linford and Oliveira) are three important plant–parasitic pests in soybean. Previous study showed that plant introduction (PI) 567516C harbored novel quantitative trait loci (QTL) conferring SCN resistance to soybean. However, QTL underlying resistance to SRKN and RN in PI 567516C remain unknown. The objectives of this study were to identify QTL for resistance to SRKN and RN in PI 567516C. Two hundred and forty-seven F_6:9_ recombinant inbred lines, derived from a cross between cultivar Magellan and PI 567516C, were evaluated for resistance to SRKN and RN. Two hundred and thirty-eight simple sequence repeats and 687 single nucleotide polymorphism markers were used to construct a genetic linkage map. Three significant QTL associated with resistance to SRKN were mapped on chromosomes (Chrs.) 10, 13 and 17. Two significant QTL associated with resistance to RN were detected on Chrs. 11 and 18. Whole-genome resequencing revealed that there might be Peking-type *Rhg1* in PI 567516C. Our study provides useful information to employ PI 567516C in soybean breeding in order to develop new cultivars with resistance to multiple nematodes.

**Electronic supplementary material:**

The online version of this article (doi:10.1007/s11032-015-0330-5) contains supplementary material, which is available to authorized users.

## Key message

We performed QTL mapping for resistance to southern root-knot nematode and reniform nematode in PI 567516C and characterized the QTL through whole-genome resequencing approach.

## Introduction

Soybean cyst nematode (SCN, *Heterodera glycine* Ichinohe), southern root-knot nematode [SRKN, *Meloidogyne incognita* (Kofoid and White) Chitwood] and reniform nematode (RN, *Rotylenchulus reniformis* Linford and Oliveira) are three important plant–parasitic pests in soybean [*Glycine max* (L.) Merrill] (Taylor and Sasser 1978; Robbins et al. 1994a; Wrather and Koenning 2006). Other than rotation with non-host crops, breeding cultivars with resistance to multiple nematode species is the most effective and environment-friendly method to control these pests (Boerma and Hussey 1992).

Considerable efforts have been made to identify sources of resistance to SRKN or RN in soybean. Luzzi et al. (1987) evaluated 2370 soybean accessions in the USDA-ARS germplasm collection from maturity groups (MG) V, VI, VII and VIII for their resistances to SRKN. Four soybean genotypes, ‘Amredo’, PI 96354, PI 408088 and PI 417444, were found to be highly resistant to SRKN (Luzzi et al. 1987). Hussey et al. (1991) and Harris et al. (2003) evaluated 139 soybean cultivars and 608 soybean accessions for resistance to SRKN, respectively. Thirty-nine soybean cultivars and seven soybean accessions were found to be resistant to SRKN (Hussey et al. 1991; Harris et al. 2003). For sources of resistance to RN, Pickett and Dyer were the first two soybean cultivars which were reported to be resistant to RN (Rebois et al. 1968). After that, more sources of resistance to RN had been identified in soybean (Robbins et al. 1994a, b; Davis et al. 1996; Robbins and Rakes 1996). It was showed that Peking-derived SCN-resistant cultivars were resistant to RN and PI 88788-derived SCN-resistant cultivars were not (Rebois et al. 1970; Robbins et al. 1994a, b; Davis et al. 1996; Robbins and Rakes 1996). Peking and PI 88788 were two major sources of resistance to SCN that have been widely deployed in soybean breeding (Concibido et al. 2004). These two soybean accessions had different functional alleles of same QTL, *Rhg1*, for resistance to SCN (Concibido et al. 2004; Meksem et al. 2001; Brucker et al. 2005; Cook et al. 2012, 2014).

In comparison with about twenty-five QTL for resistance to SCN (Concibido et al. 2004; Wang et al. 2004; Kabelka et al. 2005; Guo et al. 2005, 2006; Winter et al. 2007; Wu et al. 2009; Vuong et al. 2010), fewer QTL for resistance to SRKN or RN were mapped in soybean. Tamulonis et al. (1997) mapped two QTL with a major one on Chr. 10 and a minor one on Chr. 18 for resistance to SRKN in PI 96354. These two QTL were also confirmed in another study (Li et al. 2001). The major QTL on Chr. 10 was detected across 27 soybean cultivars with resistance to SRKN, indicating that this QTL had been widely deployed in soybean breeding (Ha et al. 2004). Recently, two independent studies reported the identification of candidate genes underlying this QTL from two different sources of resistance to SRKN, PI 96354 (Pham et al. 2013) and PI 438489B (Xu et al. 2013), respectively. Besides QTL on Chrs. 10 and 18, two other QTL were also reported to be associated with resistance to SRKN in soybean. One QTL was mapped on Chr. 7 and was responsible for resistance to SRKN race 2 in a soybean cultivar LS 5995 (Fourie et al. 2008). The other QTL was mapped on Chr. 6, which might account for a portion of genetic variation that the QTL on Chrs. 10 and 18 could not explain (Tamulonis et al. 1997; Li et al. 2001; Shearin et al. 2009).

Three major QTL responsible for resistance to RN were detected and genetically mapped on Chrs. 19, 11 and 18, respectively, in a soybean accession PI 437654 (Ha et al. 2007). Despite the fact that QTL on Chr. 19 was the largest one, combination of two QTL on Chrs. 11 and 18 was necessary and sufficient to confer high level of resistance to RN (Ha et al. 2007). These two QTLs were overlapped with the QTL for resistance to SCN (Wu et al. 2009), which provided evidences that there might be common genes responsible for resistance to SCN and RN in soybean.

Soybean accession PI 567516C was genetically unique from most sources of resistance to SCN (Arelli et al. 1997; Chen et al. 2006; Arelli et al. 2009; Vuong et al. 2010). QTL mapping for this accession identified two QTL, one on Chr. 10 and the other on Chr. 18, which were associated with resistance to SCN HG types 2.5.7, 0, 2.7, 1.3.5.6.7 and LY1 (Vuong et al. 2010). Thus, PI 567516C has great potential in breeding new soybean cultivars with resistance to SCN to fight against genetic shift of SCN populations due to long-term use of *Rhg1* and *Rhg4*, two important genes for resistance to SCN. With the aim of identifying sources of resistance to multiple nematode species, we evaluated a subset of soybean accessions with resistance to SCN for resistance to SRKN and RN. The results showed that PI 567516C not only displayed resistance to SCN but also had high level of resistance to SRKN and RN, which further made this soybean line a good source of resistance to multiple nematode species in soybean breeding. However, QTL underlying resistance to SRKN and RN in PI 567516C are still unknown, hampering the utilization of this soybean accession in developing soybean cultivars with resistance to multiple nematode species through marker-assisted selection. The objectives of this study were to identify QTL responsible for resistance to SRKN and RN in PI 567516C.

## Materials and methods

### Genetic population

A genetic population of 247 F_6:9_ recombinant inbred lines (RILs) were advanced by single seed descent method from a cross between cultivar Magellan (Schapaugh et al. 1998) and PI 567516C (Arelli et al. 1997), in 2005 at the Bradford Research and Extension Center (BREC), University of Missouri–Columbia, MO, USA. Genomic DNA was extracted from a pooled sample of leaves from five plants of each RIL following a protocol as previously described (Vuong et al. 2010).

### Southern root-knot and reniform nematode bioassays

Two hundred and forty-seven RILs and their parents, cv. Magellan and PI 567516C, were evaluated for resistance to southern root-knot nematode [SRKN, *M. incognita* (Kofoid and White) Chitwood] in the greenhouse facility at the University of Georgia, Athens, GA. A randomized complete block design (RCBD) with three replications was used for SRKN bioassays in this study. Each plant was inoculated with approximate 3000 SRKN eggs as described by Li et al. (2001). The SRKN galls that developed on each plant were counted at 36 days after inoculation.

The evaluation of the RIL mapping population for resistance to reniform nematode (RN, *R. reniformis* Linford and Oliveira) in the greenhouse follows a protocol as previously described (Robbins et al. 1999). A reproductive index (RI) was defined as the number of eggs + vermiform nematodes at test termination (Pf)/initial infestation level (Pi). This value was calculated from five replicates for each RIL and used in statistical analysis and QTL mapping.

### Statistical analysis

The SRKN gall numbers and RI among RILs were tested for normality using the PROC UNIVARIATE procedure of SAS 9.3 (SAS institute, Gary, NY, USA). A broad-sense heritability was calculated following the method described by Wu et al. (2009).

### Linkage analysis and genetic mapping

The fluorescently labeled simple sequence repeats (SSR) markers and the universal soybean linkage panel 1.0 (the USLP 1.0) containing 1536 single nucleotide polymorphism (SNP) loci (Hyten et al. 2008) were utilized to genotype the mapping population using the Illumina GoldenGate assay (Fan et al. 2006; Hyten et al. 2010). The protocol for SSR marker analysis was described by Vuong et al. (2010). Briefly, polymerase chain reaction (PCR) was conducted with a final volume of 12.5 μl on the Eppendorf 96-well master cycler gradient (Eppendorf AG, Germany). Each PCR included 40–50 ng of template DNA, 0.13 μM of labeled forward primer (Applied Biosystems, Foster City, CA, USA) and 0.2 μM of reverse primer (IDT Inc., Coralville, IA, USA), 1× reaction buffer (20 mM Tris–HCl, pH 8.0, 50 mM KCl), 2.5 mM MgCl2, 0.2 mM of each of the dNTPs and 1 unit of Taq DNA polymerase (GenScript Corp., Piscataway, NJ, USA). PCR was performed at 95 °C for 5 min, followed by 35 cycles of 94 °C for 30 s, 47 °C or 52 °C for 45 s and 72 °C for 1 min, with a final extension for 7 min at 72 °C. After purified with the Whatman PCR cleanup procedure (Whatman Inc., Piscataway, NJ, USA), PCR products were analyzed with the ABI 3100 or 3730 DNA sequencer (Applied Biosystems, Foster City, CA, USA) and the GeneMapper 3.7 program (Applied Biosystems, Foster City, CA, USA). A genetic linkage map was constructed using JoinMap 4.0 (van Ooijen 2006). A likelihood of odds (LOD) threshold score of 3.0 and a maximum genetic distance of 50 cM were used for the initial linkage grouping of markers. The soybean genetic linkage groups (LGs) (Song et al. 2004) were replaced with the new assignments of corresponding chromosome numbers (Chr.) (Grant et al. 2010).

QTL analyses were performed using the multi-QTL method (MQM) with the program MapQTL 5.0 and the appropriate cofactor (van Ooijen 2004). The threshold of LOD score was chosen to be 3.4 to declare a putative significant QTL based on the results of permutation test with 1000 runs to determine the *P* = 0.05 genome-wide significance level. The proportion of the phenotypic variance explained by the QTL effects was estimated at the QTL peaks. Additive (A) effects of significant QTL were estimated from an output of the program MapQTL 5.0. The chromosomes with LOD plots were created using the MapChart 2.2 program (Voorrips 2002).

### Whole-genome resequencing, SNP calling and copy number variation analysis

Whole-genome sequencing of cv. Magellan, Peking, PI 437654, PI 424298, cv. Hutcheson, cv. Essex, PI 567516C, PI 567387, PI 209332, PI88788, PI 567305 and PI 404198B was conducted in Beijing Genome Institute (BGI), Shenzhen, China, using Illumina technology following a described protocol (Xu et al. 2013). The sequencing depth for each sample was about 15× coverage. SNP calling was conducted by the use of SAMtools (Li et al. 2009). Copy number variation (CNV) analysis was conducted using CNV-seq software (Xie and Tammi 2009). Sequence alignment was visualized by IGV software (Thorvaldsdottir et al. 2013; Robinson et al. 2011).

## Results

### Phenotypic variation and genetic map

**Fig. 1:**
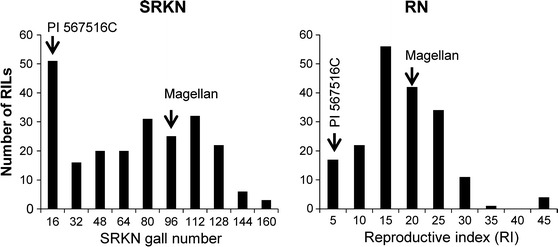
Distribution of average SRKN gall number and RN reproductive index (RI) of 247 F_6:9_ RILs developed from a Magellan × PI 567516C cross. *SRKN* southern root-knot nematode, *RN* reniform nematode. Magellan, SRKN and RN susceptible parent; PI 567516C, SRKN- and RN-resistant parent

Cultivar Magellan and PI 567516C showed significant difference in resistance to SRKN and RN (Table 1). The frequency distribution of SRKN gall number as well as RN reproductive index (RI) among 247 F_6:9_ RILs displayed large genetic variation, ranging from a low value of 0 to a high value of 156.3 and from 0.4 to 15.5, respectively (Table 1; Fig. 1). The normality test by the Shapiro–Wilk (*w*) statistic indicated that the gall number and RI were not normally distributed (Table 1; Fig. 1). The values of broad-sense heritability of gall number and RI were 0.75 and 0.32, respectively (Table 1).

**Table 1:** Summary of statistics on SRKN gall number and RN reproductive index (RI) of parental lines and 247 F_6:9_ RILs developed from a Magellan × PI 567516C cross for their responses to southern root-knot and reniform nematodes, respectively, in greenhouse assays

Trait	Magellan	PI 567516C	247 F_6:9_ RIL families				Shapiro–Wilk (*w*)	Skewness	Kurtosis	Heritability (*h*_*b*_^2^)
			Mean	Min	Max	SD				
SRKN	81.7	6.3	62.8	0	156.3	42.4	0.95 (0.0001)	0.0033	−1.16	0.75
RN	15.4	1.7	15.5	0.4	44.0	7.9	0.96 (0.0001)	0.57	1.50	0.32

*SRKN* southern root-knot nematode, *RN* reniform nematode

Two hundred and thirty-eight polymorphic SSR markers as well as 1536 SNP array were utilized for genotyping 247 RILs. Among 1536 SNP loci, six hundred and eighty-seven were found to be polymorphic between two parents. Finally, two hundred and nineteen SSR and 681 SNP markers were used to construct a linkage map, spanning approximately 2816 cM with an average distance of 3.1 cM across 20 chromosomes (Supplemental Figure 1).

### QTL associated with resistance to SRKN and RN

**Fig. 2:**
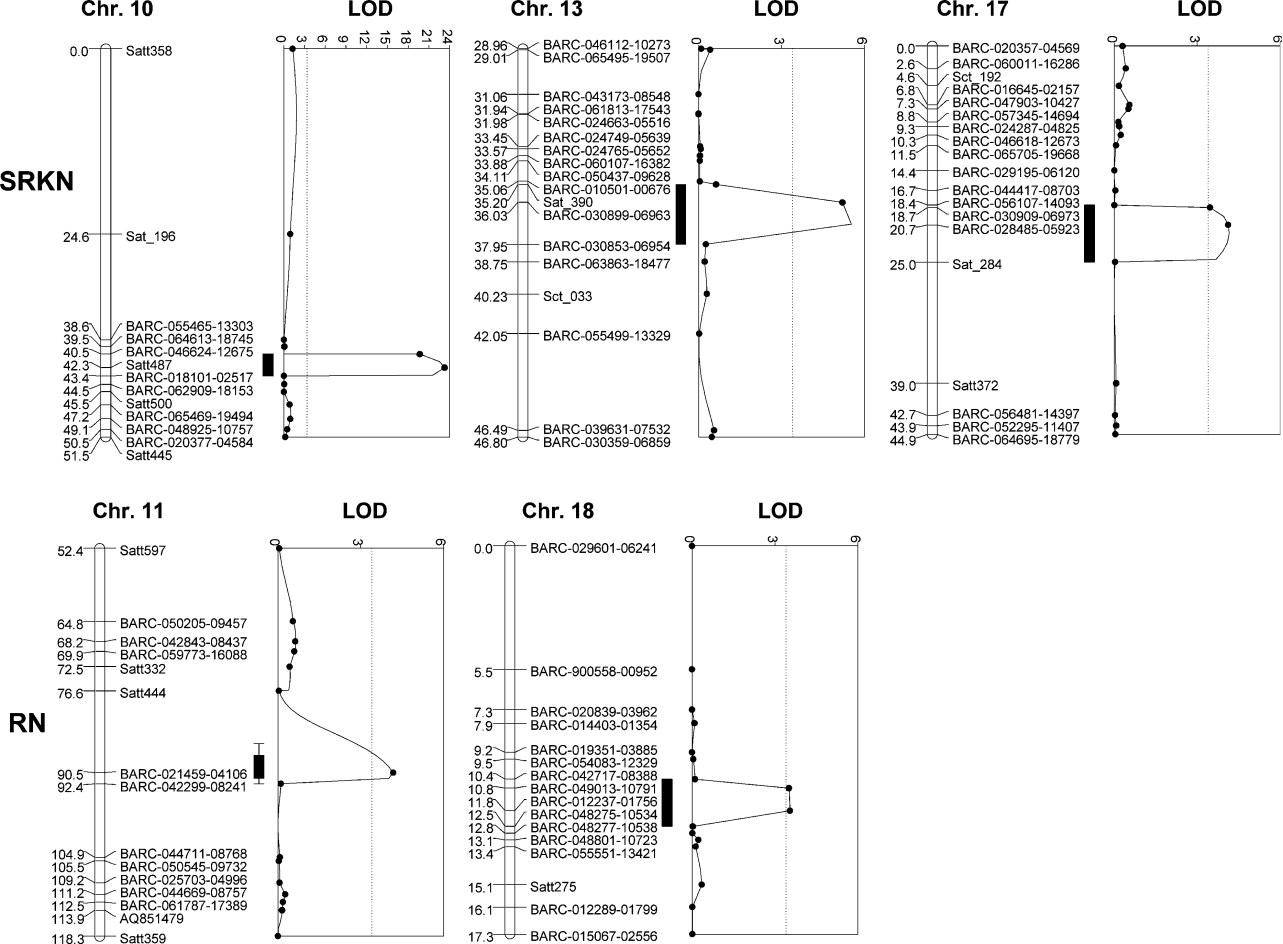
QTL likelihood plots for the SRKN gall number on Chrs. 10, 13 and 17 and RN reproductive index (RI) on Chrs. 11 and 18, respectively. LOD threshold is 3.4. The relative positions of the markers are given in centimorgan (cM). *SRKN* southern root-knot nematode, *RN* reniform nematode

Three QTL were identified to be associated with resistance to SRKN in PI 567516C (Table 2; Fig. 2). The major one with a peak LOD value of 23.3 was mapped between SNP markers BARC-046624-12675 and BARC-018101-02517 on Chr. 10, explaining 33.7 % of total phenotypic variation (Table 2; Fig. 2). The closest marker to the peak was Satt487 (Table 2). This QTL was located in a similar region to the ones in PI 96354 and PI 438489B (Tamulonis et al. 1997; Li et al. 2001; Xu et al. 2013), which indicated there might be the same QTL among these three resistant accessions. In PI 96354, four candidate genes, *Glyma10g02090* (*Glyma.10g016600*), *Glyma02100* (*Glyma.10g016700*), *Glyma10g02140* (*Glyma.10g017100*) and *Glyma10g02160* (*Glyma.10g017200*), were speculated to be responsible for resistance to SRKN (Pham et al. 2013). In PI 438489B, three candidate genes, *Glyma10g02140* (*Glyma.10g017100*), *Glyma10g02150* and *Glyma10g02160* (*Glyma.10g017200*), were identified in this QTL region (Xu et al. 2013). *Glyma10g02140* (*Glyma.10g017100*) and *Glyma10g02160* (*Glyma.10g017200*) were commonly identified in PI 438489B and PI 96354 (Pham et al. 2013; Xu et al. 2013). Both of these two genes are functional based on the most recent soybean genome assembly and annotations (Glycine max Wm82.a2.v1: http://genome.jgi.doe.gov/pages/dynamicOrganismDownload.gsf?organism=PhytozomeV10). Thus, we conducted sequence analyses of these two genes in soybean lines resistant or susceptible to SRKN through whole-genome resequencing. In addition to PI 567516C and PI 438489B, five new soybean accessions, PI 567387, PI 209332, PI 88788, PI 567305 and PI 404198B, were identified to be resistant to SRKN in our evaluation of soybean germplasm for resistance to multiple nematodes (Supplemental Table 1). These five accessions had not been reported to be resistant to SRKN before. Whole-genome resequencing was performed for these resistant accessions plus six susceptible ones (Supplemental Table 1). The results showed there were no CNVs of *Glyma10g02140* (*Glyma.10g017100*) *and Glyma10g02160* (*Glyma.10g017200*) among all tested soybean lines. However, 44 SNP/Indels in *Glyma10g02140* were commonly found in all resistant PIs in comparison with Williams 82 (Supplemental Table 1). Among these SNP/Indels, six SNPs in the first exon were non-synonymous, one of which resulted in a stop codon (Supplemental Table 1). These six SNPs were identical to the ones in PI 96354 (Pham et al. 2013). For *Glyma10g02160*, there were 11 SNP/Indels common in all the resistant PIs (Supplemental Table 1). Among these, two were located in the exon. However, neither of them results in amino acid changes.

**Table 2:** QTL associated with resistance to southern root-knot and reniform nematodes, evaluated by SRKN gall numbers and RN reproductive index (RI) on soybean roots in the Magellan × PI 567516C population

Trait	Chr. (LG)	Confidence intervals	Marker closest to the peak	Peak LOD	*R* ^2^ (%)	Addictive
SRKN	Chr. 10 (O)	BARC-046624-12675–BARC-018101-02517	Satt487	23.3	33.7	25.0
	Chr. 13 (F)	Sat_390–BARC-030853-06954	BARC-030899-06963	5.5	7.3	12.5
	Chr. 17 (D2)	BARC-056107-14093–Sat_284	BARC-028485-05923	4.2	5.1	10.1
RN	Chr. 11 (B1)	Satt444–BARC-042299-08241	BARC-021459-04106	4.2	8.9	2.8
	Chr. 18 (G)	BARC-042717-08388–BARC-048275-10534	BARC-012237-01756	3.5	7.5	2.5

*SRKN* southern root-knot nematode, *RN* reniform nematode

The other two QTL were mapped on Chrs. 13 and 17, which explained 7.3 % and 5.1 % of total phenotypic variation, respectively (Table 2; Fig. 2). QTL on Chr. 13 was previously mapped in PI 438489B (Xu et al. 2013). QTL on Chr. 17 was a novel QTL for resistance to SRKN, which has not been mapped before.

Two QTL associated with resistance to RN were mapped on Chrs. 11 and 18 in PI 567516C (Table 2; Fig. 2). The QTL on Chr. 11 was mapped between Satt444 and BARC-042299-08241. The closest marker to the QTL peak was BARC-021459-04106 (Table 2). The QTL on Chr. 18 was mapped between BARC-042717-08388 and BARC-048275-10534. The closest marker to its peak is BARC-012237-01756 (Table 2). These two QTL had been identified in PI 437654 (Ha et al. 2007).

### Characterization of *Rhg1* in PI 567516C through whole-genome resequencing method

**Fig. 3:**
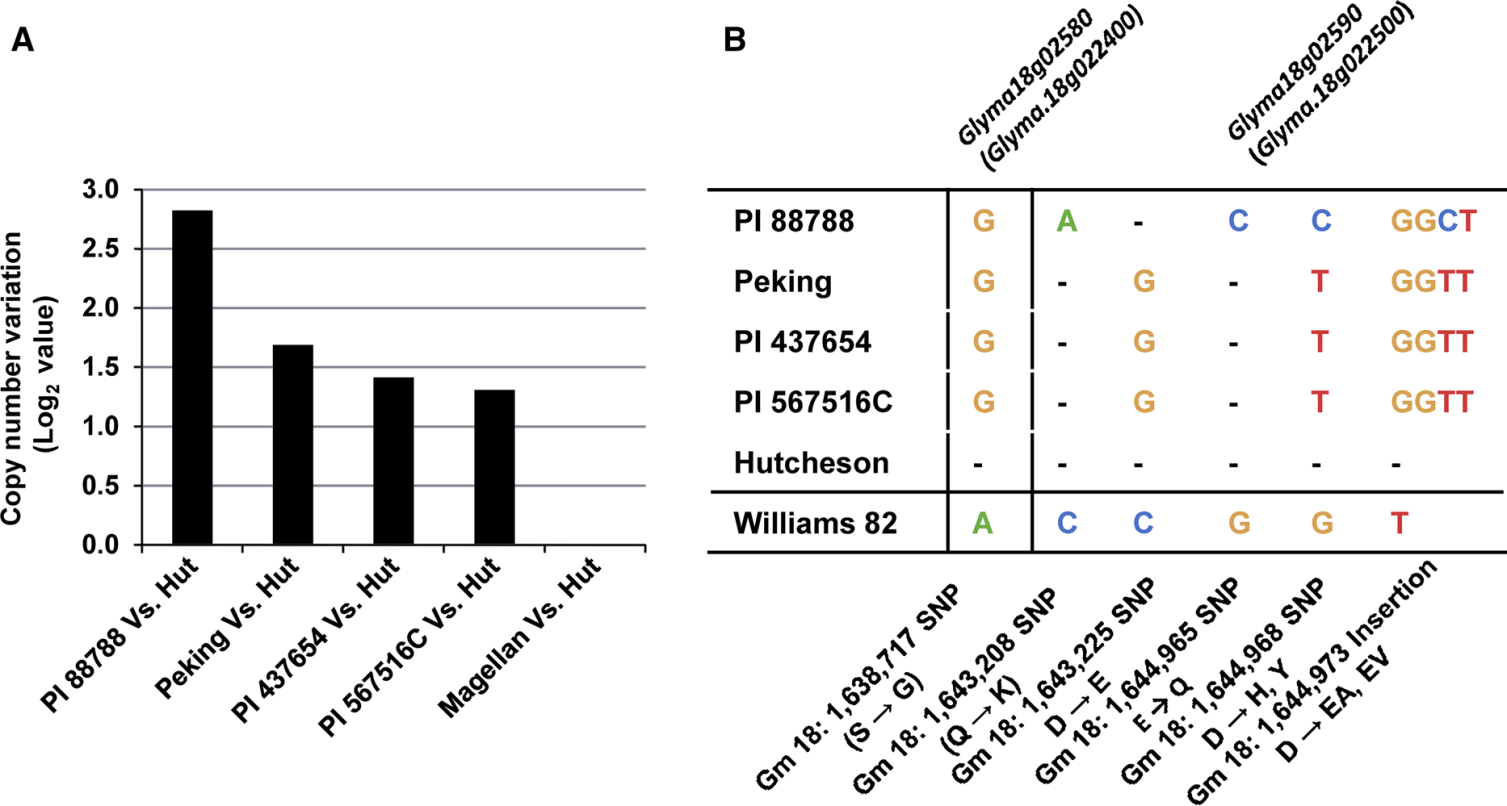
Genomic characterization of the *Rhg1* locus among PI 88788, Peking, PI 437654, PI 567516C, cv. Magellan and cv. Hutcheson using whole-genome resequencing approach. **a** Copy number variation at the *Rhg1* locus. *Hut* Hutcheson. **b** Sequence variation in the exon of *Glyma18g02590* (*Glyma.18g022500*)*. Glyma18g02590* (*Glyma.18g022500*) was predicted to encode an α-soluble NSF attachment protein (α-SNAP). The differences were highlighted with *specific colors*. *Hyphen* represents that the position is identical to Williams 82 sequence. (Color figure online)

It has been reported that there are two different *Rhg1* types, PI 88788-type and Peking-type *Rhg1* (Brucker et al. 2005; Meksem et al. 2001). Recently, copy number and sequence variation for three genes, *Glyma18g02580* (*Glyma.18g022400*), *Glyma18g02590* (*Glyma.18g022500*) and *Glyma18g02610* (*Glyma.18g022700*), between these two different types have been elucidated (Cook et al. 2012, 2014). In our study, QTL for resistance to RN on Chr. 18 in PI 567516C was located in a similar region as the *Rhg1* locus for resistance to SCN (Table 2; Fig. 2) (Concibido et al. 2004). Interestingly, a previous study on resistance to SCN did not detect the *Rhg1* locus in PI 567516C (Vuong et al. 2010). In order to determine whether PI 567516C has the *Rhg1* locus, we analyzed copy number and sequence variation of the *Rhg1* locus in PI 567516C using whole-genome resequencing approach. The results showed that PI 567516C had a similar level of CNV at the *Rhg1* locus with Peking and PI 437654, two of which had Peking-type *Rhg1* (Fig. 3a). These three soybean lines had a reduced copy number of *Rhg1* in comparison with PI 88788 (Fig. 3a). In addition to CNVs, we found that PI 567516C also had identical non-synonymous SNP/Insertions in the exon of *Glyma18g02590* (*Glyma.18g022500*) with Peking and PI 437654 (Fig. 3b). For *Glyma18g02580* (*Glyma.18g022400*), PI 567516C, PI 88788, Peking and PI 437654 had one identical SNP in the exon in comparison with Williams 82 and Hutcheson (Fig. 3b). For *Glyma18g02610* (*Glyma.18g022700*), no SNP was found among these five soybean lines. All these results indicated that there might be Peking-type *Rhg1* in PI 567516C.

## Discussion

In our study, the value of broad-sense heritability of the RI after infestation with RN was 0.32, indicating that the error variance rather than genetic variance accounted for a large part of all phenotypic variances. This might be the reason why total phenotypic variation explained by two QTL for resistance to RN in PI 567516C was small, only 16.4 % (Table 2). Large error variance might be attributed to environmental factor, which indicated that environment-controlled bioassays are warranted to reduce error variance.

Previous studies showed that the major QTL on Chr. 10 played an important role in conferring resistance to SRKN in soybean (Tamulonis et al. 1997; Li et al. 2001; Pham et al. 2013; Xu et al. 2013). In our study, this QTL was also detected and consistently mapped at the same genomic region on Chr. 10, which indicated that different sources of resistance to RKN might harbor the same gene for resistance. *Glyma10g02140* (*Glyma.10g017100*) and *Glyma10g02160* (*Glyma.10g017200*) encode pectin methylesterase inhibitors (PMEIs) that played a role in regulating the activity of pectin methylesterases (PME) in plant (Giovane et al. 2004; Pham et al. 2013). Both of these two genes were reported to be upregulated in SRKN-susceptible reaction but suppressed in SRKN-resistant reaction (Pham et al. 2013). PME is an enzyme involved in the process of cell wall breakdown. In *Arabidopsis*, loss of function of *PME3* led to higher resistance to sugar beet cyst nematode, while overexpression of *PME3* increased susceptibility (Hewezi et al. 2008). We performed sequence analyses for these two genes among SRKN-resistant and SRKN-susceptible soybean lines. The results indicated that *Glyma10g02140* (*Glyma.10g017100*) rather than *Glyma10g02160* (*Glyma.10g017200*) was more likely to be the candidate gene for QTL on Chr. 10. Besides QTL on Chr. 10, one minor QTL on Chr. 13 was identified in PI 567516C (Table 2). This QTL was also mapped in PI 438489B (Xu et al. 2013). Confirmation of this QTL in our study suggested that it was conservative and could be potentially used in soybean breeding. QTL on Chr. 17 for resistance to RN was first reported by our study. This QTL explained similar ratio of total phenotypic variation as the QTL on Chr. 13 (Table 2), which indicated that it was also an important locus for resistance to SRKN. Identification of this QTL made PI 567516C to be unique from the other two sources of SRKN resistance, PI 96354 and PI 438489B, where only two SRKN-resistant QTL were mapped (Li et al. 2001; Xu et al. 2013).

Three QTL have been reported to be associated with resistance to RN in PI 437654 (Ha et al. 2007). Among them, two QTL, one on Chr. 18 and the other on Chr. 11, were found to play critical roles in resistance to RN (Ha et al. 2007). These two QTL were also identified to be responsible for resistance to SCN in PI 437654 (Wu et al. 2009), which provided evidences that resistance to RN and SCN might concurrently share common mechanisms in soybean. QTL on Chr. 18 for resistance to RN in PI 567516C was mapped in the *Rhg1* region (Fig. 2), indicating that PI 567516C might have *Rhg1*. Through whole-genome resequencing method, we provided evidences that there might be Peking-type *Rhg1* in PI 567516C. Without *Rhg4* (Liu et al. 2012), Peking-type *Rhg1* was not able to function as a gene for resistance to SCN (Meksem et al. 2001). This might be the reason why *Rhg1* was not detected in PI 567516C in the previous study on resistance to SCN (Vuong et al. 2010). We speculated that Peking-type *Rhg1* might be in much more soybean germplasm we expected.

In a previous study, the identification of a novel QTL on Chr. 10 for resistance to SCN in PI 567516C enabled this soybean accession to be a good candidate to be used in breeding cultivars with new resistance to SCN that are different from *Rhg1-* and *Rhg4*-containing source of resistance to SCN (Vuong et al. 2010). In our study, the identification and characterization of QTL underlying resistance to RKN and RN made this PI 567516C more valuable in soybean breeding programs to develop new cultivars with broad-based resistance to multiple nematode species, not only SCN but also SRKN and RN, through marker-assisted selection.

## Electronic supplementary material

Supplementary material 1 (PDF 244 kb)

Supplementary material 2 (PDF 60 kb)
