# Supplementary material for: Identification of quantitative trait loci underlying resistance to southern root-knot and reniform nematodes in soybean accession PI 567516C
Source: Mol Breed. 2015 May 23;35(6):131. doi: 10.1007/s11032-015-0330-5 (PMC4441734; doi:10.1007/s11032-015-0330-5)
Supplement: Supplementary file 1 — Supplementary material 1 (PDF 244 kb) [file 11032_2015_330_MOESM1_ESM.pdf]

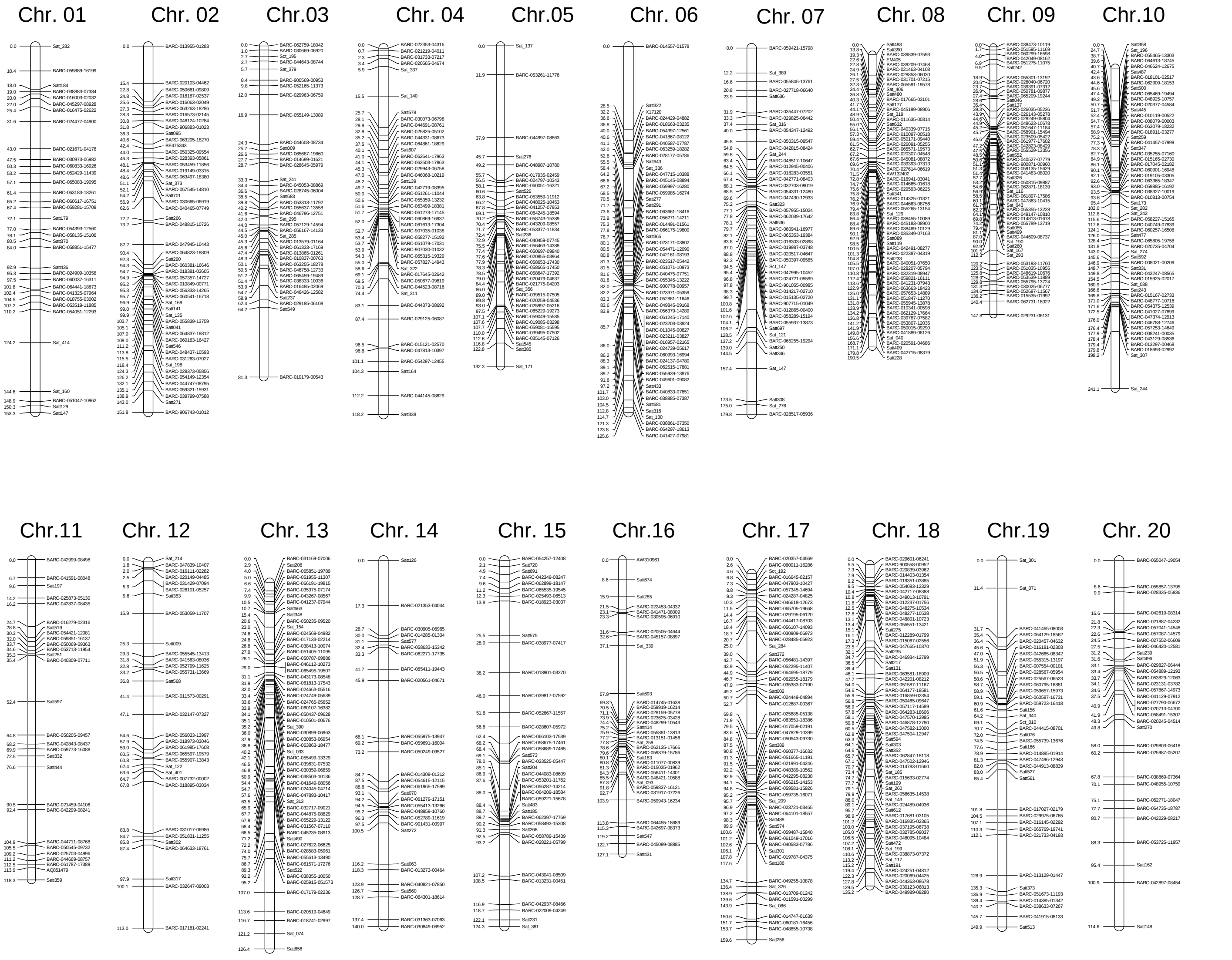

Supplemental Figure 1 Genetic linkage map constructed using a $F_{6:9}$ recombinant inbred line (RIL) population derived from a Magellan × PI 567516C cross
