# Supplementary material for: Identification of quantitative trait loci underlying resistance to southern root-knot and reniform nematodes in soybean accession PI 567516C
Source: Mol Breed. 2015 May 23;35(6):131. doi: 10.1007/s11032-015-0330-5 (PMC4441734; doi:10.1007/s11032-015-0330-5)
Supplement: Supplementary file 2 — Supplementary material 2 (PDF 60 kb) [file 11032_2015_330_MOESM2_ESM.pdf]

Supplemental Table 1 Sequence variation of *Glyma10g02140* (*Glyma.10g017100*) and *Glyma10g02160* (*Glyma.10g017200*), two candidate genes reponsible for SRKN resistance, among 13 soybean lines

Soybean line	Reaction to SRKN	*Glyma10g02140* (*Glyma.10g017100*)																																									
		First exon										Intron			2nd exon		Intron																										
		Gm 10: 1,506,154 (1,510,762)	Gm 10: 1,506,012 (1,510,620)	Gm 10: 1,505,931 (1,510,539)	Gm 10: 1,505,848 (1,510,456)	Gm 10: 1,505,843 (1,510,451)	Gm 10: 1,505,839 (1,510,447)	Gm 10: 1,505,789 (1,510,397)	Gm 10: 1,505,647 (1,510,255)	Gm 10: 1,505,584 (1,510,192)	Gm 10: 1,505,565 (1,510,173)	Gm 10: 1,505,401 (1,510,009)	Gm 10: 1,505,246 (1,509,854)	Gm 10: 1,505,086 (1,509,694)	Gm 10: 1,504,939 (1,509,694)	Gm 10: 1,504,848 (1,509,456)	Gm 10: 1,504,598 (1,509,206)	Gm 10: 1,504,384 (1,508,992)	Gm 10: 1,504,257 (1,508,865)	Gm 10: 1,504,216 (1,508,824)	Gm 10: 1,504,209 (1,508,817)	Gm 10: 1,504,159 (1,508,767)	Gm 10: 1,504,125 (1,508,733)	Gm 10: 1,504,086 (1,508,694)	Gm 10: 1,504,012 (1,508,620)	Gm 10: 1,503,953 (1,508,561)	Gm 10: 1,503,941 (1,508,549)	Gm 10: 1,503,791 (1,508,399)	Gm 10: 1,503,777 (1,508,385)	Gm 10: 1,503,774 1,508,382)	Gm 10: 1,503,762 (1,508,370)	Gm 10: 1,503,728 (1,508,336)	Gm 10: 1,503,636 (1,508,244)	Gm 10: 1,503,606 (1,508,214)	Gm 10: 1,503,567 (1,508,175)	Gm 10: 1,503,372 (1,507,980)	Gm 10: 1,503,353 (1,507,961)	Gm 10: 1,503,326 (1,507,934)	Gm 10: 1,503,311 (1,507,919)	Gm 10: 1,503,099 (1,507,707)	Gm 10: 1,503,016 (1,507,624)	Gm 10: 1,502,971 (1,507,579)	Gm 10: 1,502,926 (1,507,534)
Williams 82	s	G	C	C	A	A	G	C	T	A	G	T	A	A	A	G	CTGTTG<br>AAAATA<br>GTCCTA<br>CATTGG<br>ATGTTT<br>[illegible]	TCT	T	A	GT	G	AC	A	A	ATCTTT<br>TTTCA<br>GCATT<br>ATGTT<br>CAATC<br>[illegible]	C	G	C	TC	G	T	C	C	C	C	T	C	A	G	ATAA	G	G
PI 437654	S	-	-	-	-	-	-	-	-	-	-	-	-	-	-	-	-	-	-	-	-	-	-	-	-	-	-	-	-	-	-	-	-	-	-	-	-	-	-	-	-	-	-
Magellan	S	-	-	-	-	-	-	-	-	-	-	-	-	-	-	-	-	-	-	-	-	-	-	-	-	-	-	-	-	-	-	-	-	-	-	-	-	-	-	-	-	-	-
PI 424298	S	-	-	-	-	-	-	-	-	-	-	-	-	-	-	-	-	-	-	-	-	-	-	-	-	-	-	-	-	-	-	-	-	-	-	-	-	-	-	-	-	-	-
Hutcheson	S	-	-	-	-	-	-	-	-	-	-	-	-	-	-	-	-	-	-	-	-	-	-	-	-	-	-	-	-	-	-	-	-	-	-	-	-	-	-	-	-	-	-
Essex	S	-	-	-	-	-	-	-	-	-	-	-	-	-	-	-	-	-	-	-	-	-	-	-	-	-	-	-	-	-	-	-	-	-	-	-	-	-	-	-	-	-	-
PI 438489B	R	A	T* (V^{93}I)	A* (D^{120}Y)	C	C* (L^{149}R)	A	T* (C^{167}Y)	A* (E^{214}D)	G	A* (Stop)	A	AAAAA	G	C	T	C	T	A	G	G	A	A	C	G	A	T	C	A	T	A	C	G	T	A	T	G	CCTCTGCC	G	A	A	A	A
PI 567516C	R	A	T* (V^{93}I)	A* (D^{120}Y)	C	C* (L^{149}R)	A	T* (C^{167}Y)	A* (E^{214}D)	G	A* (Stop)	A	AAAAA	G	-	T	C	T	A	G	G	A	A	C	G	A	T	C	A	T	A	C	G	T	A	T	G	CCTCTGCC	G	A	A	A	A
PI 567387	R	A	T* (V^{93}I)	A* (D^{120}Y)	C	C* (L^{149}R)	A	T* (C^{167}Y)	A* (E^{214}D)	G	A* (Stop)	A	AAAAA	G	-	T	C	T	A	G	G	A	A	C	G	A	T	C	A	T	A	C	G	T	A	T	G	CCTCTGCC	G	A	A	A	A
PI 209332	R	A	T* (V^{93}I)	A* (D^{120}Y)	C	C* (L^{149}R)	A	T* (C^{167}Y)	A* (E^{214}D)	G	A* (Stop)	A	AAAAA	G	-	T	C	T	A	G	G	A	A	C	G	A	T	C	A	T	A	C	G	T	A	T	G	CCTCTGCC	G	A	A	A	A
PI88788	R	A	T* (V^{93}I)	A* (D^{120}Y)	C	C* (L^{149}R)	A	T* (C^{167}Y)	A* (E^{214}D)	G	A* (Stop)	A	AAAAA	G	C	T	C	T	A	G	G	A	A	C	G	A	T	C	A	T	A	C	G	T	A	T	G	CCTCTGCC	G	A	A	A	A
PI 567305	R	A	T* (V^{93}I)	A* (D^{120}Y)	C	C* (L^{149}R)	A	T* (C^{167}Y)	A* (E^{214}D)	G	A* (Stop)	A	AA	G	-	T	C	T	A	G	-	A	A	C	-	A	T	C	A	T	A	C	G	T	A	T	G	CCTCTGCC	G	A	A	A	A
PI 404198B	R	A	T* (V^{93}I)	A* (D^{120}Y)	C	C* (L^{149}R)	A	T* (C^{167}Y)	A* (E^{214}D)	G	A* (Stop)	A	AA	G	-	T	C	T	A	G	-	A	A	C	-	A	T	C	A	T	A	C	G	T	A	T	G	CCTCTGCC	G	A	A	A	A

Note:

1. Evaluation of Mi resistance for the above soybean lines were conducted for at least two replicates. S, susceptible; R, resistant.
2. - represent that the position is identical to Williams 82 sequence.
3. The positions highlighted with red color represent the exon.
4. The SNP/Indels highlighted with yellow color represent the sequence variation common in all resistant lines but different from all susceptible lines.
5. The SNPs marked with * in the exon regions represent nonsynonymous mutation. For other SNPs without * represent synonymous mutation.

						Glyma10g02160 (*Glyma.10g017200*)																																						
					**3rd exon**	**Promoter**							**5' UTR**	**First exon**								**Intron**																			**Last exon**		**3' UTR**	
Gm 10: 1,502,811 (1,507,419)	Gm 10: 1,502,796 (1,507,404)	Gm 10: 1,502,789 (1,507,397)	Gm 10: 1,502,748 (1,507,356)	Gm 10: 1,502,699 (1,507,307)	Gm 10: 1,502,515 (1,507,123)	Gm 10: 1,511,592 (1,518,544)	Gm 10: 1,511,599 (1,518,551)	Gm 10: 1,511,724 (1,518,676)	Gm 10: 1,511,745 (1,518,697)	Gm 10: 1,511,809 (1,518,761)	Gm 10: 1,511,816 (1,518,768)	Gm 10: 1,511,898 (1,518,850)	Gm 10: 1,512,433 (1,519,385)	Gm 10: 1,512,614 (1,519,566)	Gm 10: 1,512,905 (1,519,857)	Gm 10: 1,512,938 (1,519,890)	Gm 10: 1,513,052 (1,520,004)	Gm 10: 1,513,162 (1,520,114)	Gm 10: 1,513,164 (1,520,116)	Gm 10: 1,513,186 (1,520,138)	Gm 10: 1,513,382 (1,520,334)	Gm 10: 1,513,614 (1,520,566)	Gm 10: 1,513,646 (1,520,598)	Gm 10: 1,513,716 (1,520,668)	Gm 10: 1,513,738 (1,520,690)	Gm 10: 1,513,779 (1,520,731)	Gm 10: 1,513,865 (1,520,817)	Gm 10: 1,513,869 (1,520,821)	Gm 10: 1,513,871 (1,520,823)	Gm 10: 1,513,876 (1,520,828)	Gm 10: 1,513,919 (1,520,871)	Gm 10: 1,513,936 (1,520,888)	Gm 10: 1,513,949 (1,520,901)	Gm 10: 1,513,968 (1,520,920)	Gm 10: 1,514,124 (1,521,076)	Gm 10: 1,514,160 (1,521,112)	Gm 10: 1,514,162 (1,521,114)	Gm 10: 1,514,260 (1,521,212)	Gm 10: 1,514,261 (1,521,213)	Gm 10: 1,514,281 (1,521,233)	Gm 10: 1,514,707 (1,521,659)	Gm 10: 1,514,717 (1,521,669)	Gm 10: 1,515,049 (1,522,001)	Gm 10: 1,515,053 (1,522,005)
G	T	C	A	C	G	G	C	A	C	A	T	AAATATATA	A	A	T	A	A	G	A	T	T	GA	C	TTAT	A	A	A	T	A	A	T	AATTAAAATAAA	A	A	C	G	T	G	AA	A	A	G	G	C
-	-	-	-	-	-	-	-	-	-	-	TT	-	-	-	-	-	-	-	-	-	-	-	-	-	-	-	-	-	-	-	-	-	-	-	-	-	-	-	-	-	-	-	-	-
-	-	-	-	-	-	-	-	-	-	-	TT	-	-	-	-	-	-	-	-	-	-	-	-	-	-	-	-	-	-	-	-	-	-	-	-	-	-	-	-	-	-	-	-	-
-	-	-	-	-	-	-	-	-	-	-	TT	-	-	-	-	-	-	-	-	-	-	-	-	-	-	-	-	-	-	-	-	-	-	-	-	-	-	-	-	-	-	-	-	-
-	-	-	-	-	-	-	-	-	-	-	-	-	-	-	-	-	-	-	-	-	-	-	-	-	-	-	-	-	-	-	-	-	-	-	-	-	-	-	-	-	-	-	-	-
-	-	-	-	-	-	-	-	-	-	-	-	-	-	-	-	-	-	-	-	-	-	-	-	-	-	-	-	-	-	-	-	-	-	-	-	-	-	-	-	-	-	-	-	-
C	C	T	T	T	A	-	-	AA	G	-	-	A	-	T	C	C	-	C*(R^{218}T)	-	-	-	G	G	TT	G	-	G	-	-	AA	A	A	-	T	T	A	A	-	A	G	T	A*(D^{457}N)	A	A
C	C	T	T	T	A	T	G	AA	G	-	-	A	-	T	C	C	-	C*(R^{218}T)	-	-	-	G	G	TT	G	-	G	-	-	AA	A	A	-	T	T	A	A	-	A	G	T	A*(D^{457}N)	A	A
C	C	T	T	T	A	T	G	AA	G	GT	-	A	-	T	C	C	-	C*(R^{218}T)	-	-	-	G	G	TT	G	-	G	-	-	AA	A	A	-	T	T	A	A	-	A	G	T	A*(D^{457}N)	A	A
C	C	T	T	T	A	T	G	AA	G	GT	-	A	-	T	C	C	-	C*(R^{218}T)	-	-	-	G	G	TT	G	-	G	-	-	AA	A	A	-	T	T	A	A	-	A	G	T	A*(D^{457}N)	A	A
C	C	T	T	T	A	T	G	AA	G	GT	-	A	-	T	C	C	-	C*(R^{218}T)	-	-	-	G	G	TT	G	-	G	-	-	AA	A	A	-	T	T	A	A	-	A	G	T	A*(D^{457}N)	A	A
C	C	T	T	T	A	-	-	AA	G	-	TT	AATAT	C	-	C	C	C	-	G* (T^{219}A)	C*(V^{226}A)	C	-	-	-	-	G	-	A	T	-	-	-	C	-	-	-	-	GA	-	G	-	-	A	A
C	C	T	T	T	A	-	-	AA	G	-	TT	AATAT	-	-	C	C	C	-	G* (T^{219}A)	C*(V^{226}A)	C	-	-	-	-	G	-	A	T	-	-	-	C	-	-	-	-	GA	-	G	-	-	A	A

Gm 10: 1,515,070 (1,522,022)	Gm 10: 1,515,085 (1,522,037)	Gm 10: 1,515,087 (1,522,039)	Gm 10: 1,515,089 (1,522,041)	Gm 10: 1,515,234 (1,522,186)
C	G	C	T	A
-	-	-	-	-
-	-	-	-	-
-	-	-	-	-
-	-	-	-	-
-	-	-	-	-
G	A	G	G	-
G	A	G	G	-
G	A	G	G	-
G	A	G	G	-
G	A	G	G	-
G	A	G	G	AA
G	A	G	G	AA
